# Enhanced drug delivery to cancer cells through a pH-sensitive polycarbonate platform†

**DOI:** 10.1039/d2bm01626e

**Published:** 2022-12-15

**Authors:** Maria C. Arno, Joshua D. Simpson, Lewis D. Blackman, Ruairí P. Brannigan, Kristofer J. Thurecht, Andrew P. Dove

**Affiliations:** a School of Chemistry, University of Birmingham Edgbaston Birmingham B15 2TT UK m.c.arno@bham.ac.uk a.dove@bham.ac.uk; b Institute of Cancer and Genomic Sciences, University of Birmingham Edgbaston Birmingham B15 2TT UK; c Australian Institute for Bioengineering and Nanotechnology, The University of Queensland St. Lucia Queensland 4072 Australia; d Centre for Advanced Imaging, The University of Queensland St. Lucia Queensland 4072 Australia; e ARC Centre of Excellence in Convergent Bio-Nano Science and Technology, The University of Queensland St. Lucia Queensland 4072 Australia; f Department of Chemistry, The University of Warwick Coventry CV4 7AL UK

## Abstract

Polymer–drug conjugates are widely investigated to enhance the selectivity of therapeutic drugs to cancer cells, as well as increase circulation lifetime and solubility of poorly soluble drugs. In order to direct these structures selectively to cancer cells, targeting agents are often conjugated to the nanoparticle surface as a strategy to limit drug accumulation in non-cancerous cells and therefore reduce systemic toxicity. Here, we report a simple procedure to generate biodegradable polycarbonate graft copolymer nanoparticles that allows for highly efficient conjugation and intracellular release of *S*-(+)-camptothecin, a topoisomerase I inhibitor widely used in cancer therapy. The drug–polymer conjugate showed strong efficacy in inhibiting cell proliferation across a range of cancer cell lines over non-cancerous phenotypes, as a consequence of the increased intracellular accumulation and subsequent drug release specifically in cancer cells. The enhanced drug delivery towards cancer cells *in vitro* demonstrates the potential of this platform for selective treatments without the addition of targeting ligands.

## Abstract 2

In this paper we a report a pH-sensitive polycarbonate conjugated with the anticancer drug camptothecin that shows enhanced drug delivery towards cancer cells.

## Graphical abstract

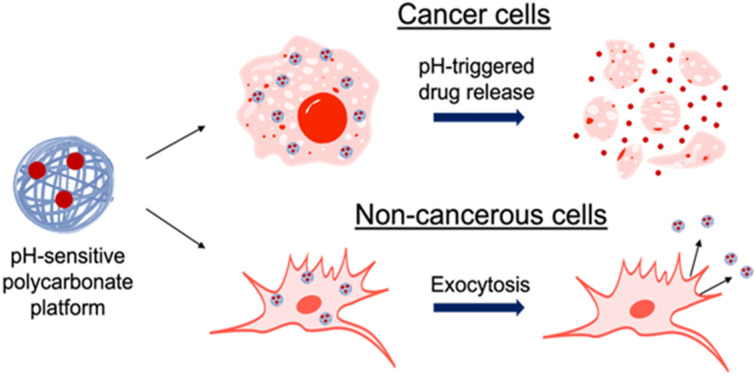

## Introduction

Despite the large number of anticancer drugs on the market, a lack of solubility and delivery strategies eliminates promising therapeutic candidates from the clinical development pipeline. Among the biggest pitfalls candidate chemotherapeutics face are limited specificity for the target, insufficient cellular uptake, and adverse side effects due to activity in healthy cells and tissues. Drug–polymer conjugates have been explored as drug delivery vehicles owing to their ability to increase the circulation time of cytotoxic drugs in the blood, improve solubility, minimize immune recognition and uptake by reticuloendothelial system, and also enhance structural stability of fragile cargo.^1–5^ Among them, successful formulations such as Doxil (pegylated liposome-encapsulated doxorubicin) and Abraxane (albumin bound paclitaxel) have reached the market,^6^ while three other polymer-camptothecin conjugates MAG-CPT (methacryloylglycynamide), Prothecan (poly(ethylene)glycol), and CRLX101 (cyclodextrin-poly(ethylene)glycol) are now in Phase I and II of clinical trials, respectively.^7–11^ Nanoparticles incorporating cytotoxic drugs can either accumulate in tumour sites *via* passive^12,13^ or active targeting, the latter being mediated by a ligand specific for surface molecules of certain cancerous cells.^14–17^ Although active targeting has the advantage of being able to selectively bind specific receptors on the cancer cells’ surface, passive targeting can be regarded as a more general strategy, addressing a wide spectrum of solid tumours with leaky vasculature.^18–23^ Moreover, the absence of targeting agents makes these drug–polymer conjugates easier to synthesise, cheaper, more scalable, and with longer storage shelf-life due to the absence of a biologically degradable molecule. However, their lack of selectivity for cancer cells often makes them a less attractive choice as delivery systems. Recently, an interest in how physicochemical properties of materials can interact with surface molecules, lipid membranes, and metabolic processes, has begun to shed insight into how these materials could be designed to exhibit improved selectivity and uptake without the need for attachment of biomolecules or targeting motifs. Combined with effective stimuli-responsive linker chemistry, an exciting new niche of polymer–drug conjugates could soon revitalize previously failed chemotherapeutic candidates.

While a number of polymeric nanoparticles have been specifically designed to respond to extracellular stimuli, intracellular stimuli are equally important. For example, pH sensitive nanoparticles have been designed to incorporate cleavable linkers that can be triggered to release cargoes in endosomes or lysosomes where pH values can drop as low as 4.0.^24–29^ Among these, hydrazone,^30–35^ orthoester,^36–39^ vinyl ether,^40–42^ cys-acotinyl,^43–45^ acetal,^46–51^ and Schiff base^52,53^ have been widely reported to achieve systematic nanoparticle disassembly. While the presence of pH-responsive units facilitates intracellular drug release, nanoparticles containing these linkers are normally indiscriminately uptaken by cancerous cells as well as non-cancerous cells, with the risk of generating substantial systemic toxicity.

Recently, we reported the synthesis of a polycarbonate scaffold (poly(NTC)) containing both a versatile norbornene functionality for post-polymerisation modification and a pH-sensitive acetal group for triggered release.^51^ In contrast to approaches in which drugs are encapsulated within micelles, covalent attachment of the drug to the polymeric carrier represents a more attractive strategy as it allows predictable drug loading, enhanced stability at physiological conditions, and a high level of control over the release event, which leads to enhanced therapeutic efficacy and minimizes potential side effects.^27^ In this work, we functionalise PNTC with an anticancer drug, camptothecin, and investigate the ability of the drug–polymer conjugate to form stable nanoparticles that can be internalized by a variety of cancer cell lines and inhibit their proliferation. We then demonstrate that the camptothecin-conjugated system can predominantly affect the proliferation of cancer cells *vs.* non-cancerous cell lines, without the need of targeting agents.

## Results & discussion

The functional monomer used in this work, 9-norbornene-2,4,8,10-tetraoxaspiro[5,5]undecan-3-one (NTC), was synthesized as previously reported.^51^ The ring opening polymerisation (ROP) of NTC catalysed by 1,8-diazabicyclo[5.4.0]undec-7-ene (DBU) was initiated from benzyl alcohol in CHCl_3_ at ambient temperature. The resultant polymer exhibited a narrow molar mass distribution (*Đ*_M_ = 1.15) and a degree of polymerisation (DP) of 20, with retention of the norbornene functionality (Fig. S1 and S2).†

**Scheme 1:**
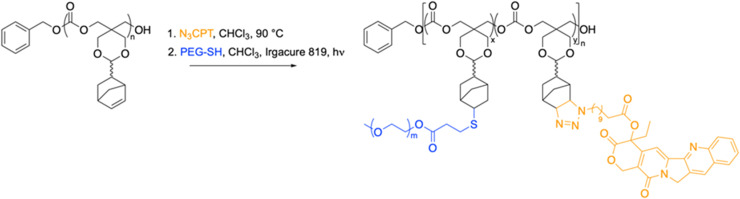
Synthesis of PNTC-*g*-CTP-*g*-PEG from PNTC.

We have shown that poly-NTC (PNTC) can undergo post-polymerisation modifications through a variety of chemistries, including Huisgen 1,3-dipolar cycloaddition, inverse electron demand Diels–Alder (DA_inv_) reaction, and photoinduced radical thiol–ene addition.^51^ In order to conjugate CPT to the polymer backbone *via* the norbornene moiety, the synthesis of a thiol-functionalised version of the drug was firstly attempted. However, as a consequence of the high reactivity of the thiol group, a pure thiol-functional CPT derivative was not achieved in reasonable yields. Nevertheless, the versatility of our polymer system allowed us to explore different functionalization pathways. Following a reported procedure,^54,55^ the hydroxyl group in position 20 on the E ring was esterified with 11-azidoundecanoic acid, to present an azide functional group which could readily react with the norbornene on the polymer chain (Scheme 1, Fig. S3–S5).†

Drug loading was calculated by integration of the ^1^H NMR spectroscopy resonances at *δ* = 8.42–7.68 and *δ* = 6.15–5.93 that correspond to camptothecin and the norbornene alkene protons from the polymer backbone, respectively. As expected, the targeted 15% conjugation was successfully achieved, indicating an average of 3 units of camptothecin are present per polymer chain (Fig. S5†). SEC analysis also confirmed full attachment of the drug to the polymer backbone, showing overlapping of the RI signal with the UV signal at 375 nm attributed to camptothecin (Fig. S6).†

In order to efficiently serve as a drug delivery carrier, the polymer conjugate should be able to self-assemble into nanostructures that isolate the drug from the external environment and deliver it intracellularly. For this reason, an amphiphilic copolymer was synthesized by the introduction of poly(ethylene glycol) (PEG) to the polymer backbone *via* the norbornene functionality. 3-Mercaptopropionate-functionalised poly(ethylene glycol) methyl ether (*M*_n_ = 550 g mol^−1^) (PEG-SH) was grafted to PNTC-CPT (DP20) using a radical thiol–ene addition (Fig. S6 and S7).†

**Fig. 1:**
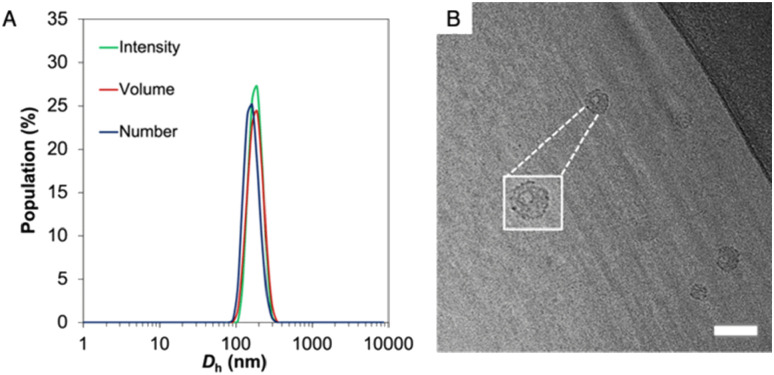
(A) Dynamic light scattering analysis and (B) representative cryo-TEM image of PNTC-*g*-CPT-*g*-PEG nanoparticles (inset = zoom-in of one particle). Particle size was found to be 130 nm. The hollow structure of the nanoparticles is evident from the TEM picture. Scale bar equals to 100 nm.

The resulting amphiphilic drug–polymer conjugate was able to self-assemble into spherical nanoparticles of 130 nm in diameter, as confirmed by multi-angle dynamic light scattering (DLS) and static light scattering (SLS) analyses, as well as through transmission electron microscopy (TEM) and cryo-EM (Fig. 1, S8 and S9).†

Importantly, while dry state TEM and DLS cannot give information about the shape of the nanoparticles, the hollow structure was confirmed by both cryo-EM and SLS (*R*_g_/*R*_h_ = 0.992). Beyond the versatility in the conjugation approach, a second advantage of PNTC is represented by the pH-sensitive acetal bond.^48,49^ The nanoparticles’ stability at acidic pH was investigated by multi-angle light scattering (MALS). After incubation at pH 5.0 and 4.0 for 12 h the *R*_g_/*R*_h_ ratio and *N*_agg_ increased considerably, which suggests a change from a hollow structure at pH 7.4 (*R*_g_/*R*_h_ = 0.99) to a swelled and elongated structure at acidic pH (*R*_g_/*R*_h_ > 2) (Table S1†). This indicated that the particles were stable at pH 6 but responded to the external environment (pH ≤ 5) with a structural change, within a 12 h window. This observation is in agreement with the data reported by Chen *et al.*, for their PEG conjugated poly mono-2,4,6-trimethoxybenzylidene-pentaerythritol carbonate (PEG-PTMBPEC) block copolymer and with our previous work with poly(NTC).^23,51,56^ Although an ester linker is also present between camptothecin and the polymer backbone, acetals hydrolyse much faster at pH 5 or lower as well as in cellular endosomes and lysosomes.^47,57^ Hence it is expected that insignificant ester hydrolysis occurs within the time frames considered. It has also been reported that hydroxy polycarbonate derived from pentaerythritol is highly hydrophilic but not soluble in water,^58^ which may explain the preservation of micellar structures even after complete acetal hydrolysis.

**Fig. 2:**
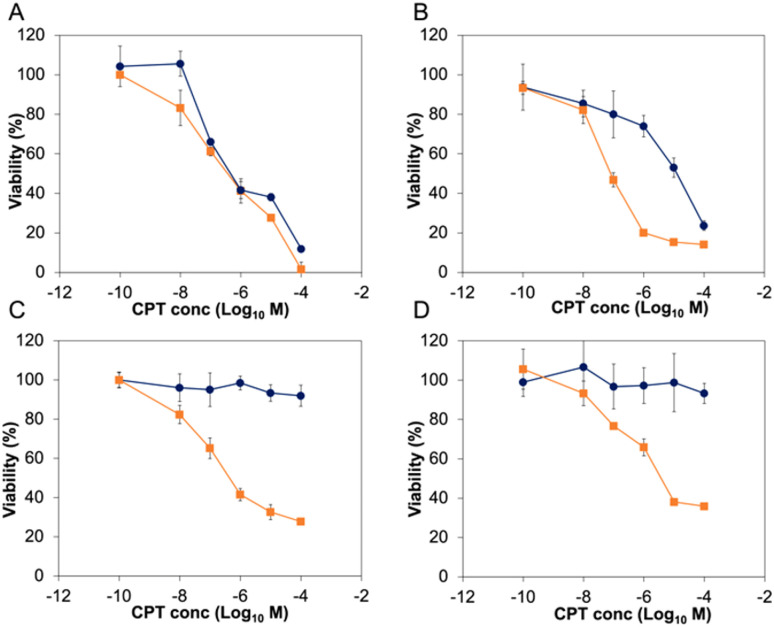
Viability of A549 (A) and PC3 (B) cancer cell lines, and IMR-90 (C), and HS792 (D) non-cancerous cell lines when incubated for 72 h with camptothecin azide (orange line, squared marker), PNTC-*g*-CPT-*g*-PEG (dark blue line, round marker).

Before exploring the biological activity of the PNTC-*g*-CPT-*g*-PEG nanoparticles, their stability in cell culture conditions was investigated by suspending the nanostructures in Dulbecco's Modified Eagle Medium (DMEM) containing 10% fetal bovine serum (FBS) for 72 h. Analysis by DLS demonstrated that the nanoparticles are colloidally stable in these conditions, without significant change in size or morphology (Fig. S10†). Subsequently, the cytotoxicity of the PNTC-*g*-CPT-*g*-PEG nanoparticles was evaluated using a series of cancer cell lines: A549 (lung cancer epithelial cells), PC3 (prostate cancer epithelial cells), MCF-7 (breast cancer epithelial cells), and MDA-MB-468 (breast cancer epithelial cells).^59^ Cells were incubated with a concentration range (0–100 μM) of drug–polymer conjugate or free camptothecin azide for a period of 72 h. Metabolic activity was then measured using the MTS metabolic assay. Proliferation of all cancerous cell lines was significantly decreased by incubation with the CPT-loaded nanoparticles (Fig. 2 and S11†). In particular, A549 cells were the most affected with an IC_50_ of 3.06 μM, followed by MDA-MB-468 (3.82 μM), PC3 (4.48 μM), and MCF-7 (6.29 μM), with a *p*-value ≤ 0.05, which was calculated using a student *t*-test. Full IC50 values and 95% confidence intervals are available in the ESI (Tables S2 and S3).†

As expected, the PNTC-*g*-PEG grafted copolymer is not toxic to any of the cell lines investigated, demonstrating the decrease in cell viability can be only ascribed to the cytotoxicity of camptothecin (Fig. S12†). Predictably, camptothecin alone (even in the azide-modified form) is more toxic compared to the drug–polymer conjugate (A549: 0.184 μM, MCF-7: 0.44 μM, MDA-MB-468: 0.082 μM, PC3: 0.068 μM, p ≤ 0.05). This is not uncommon for drug–polymer systems, and in this case is likely to be a consequence of the fact that camptothecin needs first to be released from the polymer backbone before entering the cell nucleus and subsequently inducing DNA damage through interference with its Topoisomerase target.

In sharp contrast to these results, the same concentration of nanoparticles did not affect the proliferation of the four non-cancerous cell lines tested (Fig. 2 and S11†). Indeed, IMR-90 (human lung fibroblasts), HS792 (human fibroblasts), CHO-K1 (hamster ovarian cells), and NIH-3T3 (murine fibroblasts) reported a viability higher than 80% even at the highest CPT concentration tested (100 μM). On the other hand, free camptothecin remarkably affected the proliferation of all non-cancerous cell lines tested (Fig. 2 and S11, Table S3†). The significant decrease of proliferation of cancer cell lines was surprising, especially considering there is no appreciable difference between the intracellular pH of most non-cancerous and cancerous cell lines.^60^ However, it can be expected to observe a higher nanoparticle uptake in cancer cells, and hence a more significant decrease in viability, simply as a result of their faster metabolism.

**Fig. 3:**
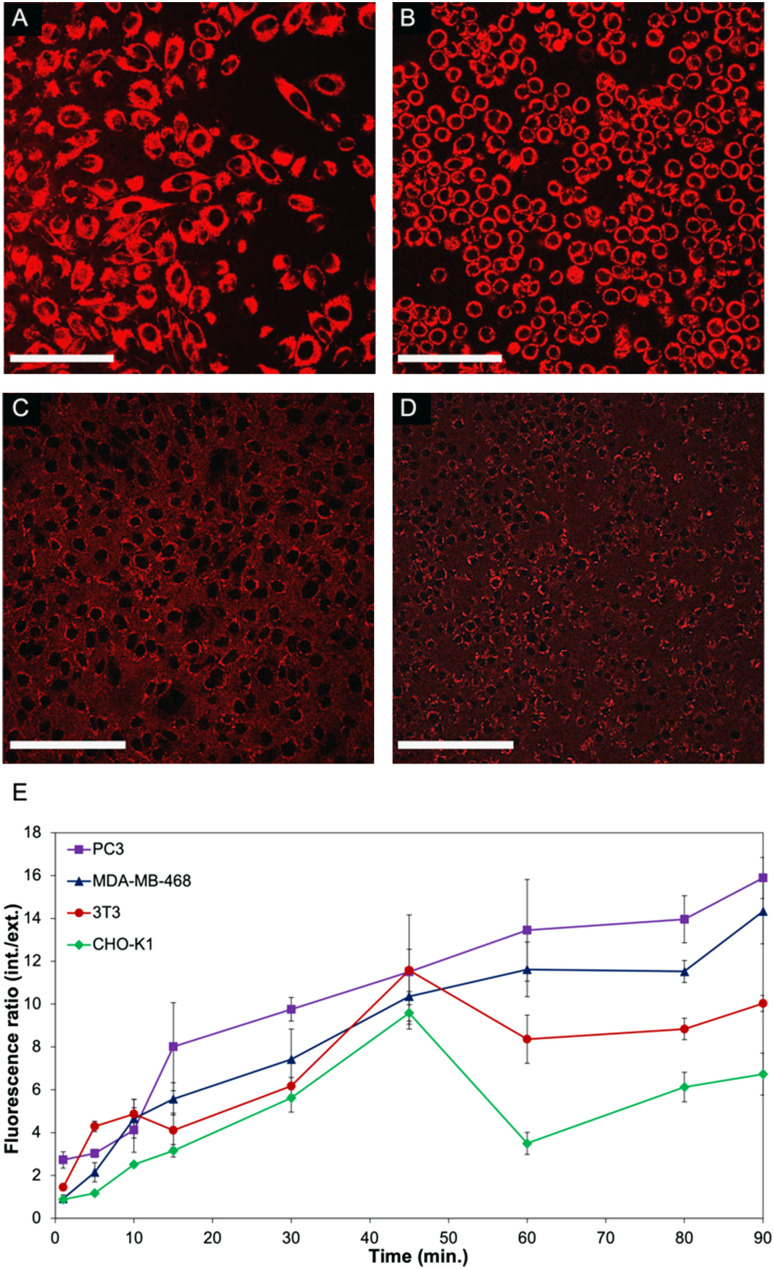
Confocal fluorescent images of PC3 (A), MDA-MB-468 (B), 3T3 (C), and CHO-K1 (D) cells incubated with PNTC-*g*-CPT-*g*-Cy5-*g*-PEG for 90 minutes. Scale bar = 10 μm. (E) Fluorescence ratio over time obtained by measuring fluorescent intensity from points inside and outside cells. A minimum of 10 points were chosen each side per time point.

In order to further investigate the mechanism that leads to such a significant difference in the viability of non-cancerous cells compared to cancer cell lines, live cell experiments were performed. To facilitate monitoring of the polymer-CPT conjugate inside cells using confocal fluorescence microscopy, PNTC was functionalised with a fluorescent dye (Cy5-azide), as well as camptothecin and PEG (Fig. S13–S15†). Nanoparticle internalization was monitored by measuring the fluorescence intensity inside and outside the cell over time, using the red fluorescent channel for Cy5 (Fig. 3 and S16, S17†). Confirming our results, a distinct behaviour was observed between cancerous and non-cancerous cell lines. Indeed, cancer cells (PC3 and MDA-MB-468 were selected in this experiment) constantly uptake the drug–polymer conjugate over time, increasing the concentration of camptothecin inside the cell, which eventually leads to cell death (Fig. 3A, B and E). On the contrary, the same experiment performed with non-cancerous cells shows that 3T3 and CHO-K1 cells initially uptake the nanoparticles but then excrete the material from their cytoplasm, hence decreasing the overall concentration of free camptothecin inside the cell (Fig. 3C–E). While the live experiment was conducted for 90 minutes, confocal images of 3T3 and CHO-K1 cells after 24 hours still show the drug–polymer conjugate predominantly outside of the cells, as is evidenced from the high extracellular fluorescence (Fig. S16†). This mechanism, exocytosis, is well known and is particularly efficient in non-cancerous cells, which have better mechanisms to recognize and excrete toxic molecules, impaired vesicular trafficking being a common hallmark of cancerous cells. While camptothecin alone cannot be excreted from non-cancerous cells, and as a result induces cell death (Fig. 2 and S11†), the drug–polymer conjugate is easily expelled from the cell cytoplasm, thus not affecting cell viability. Indeed, this behaviour was also observed when only Cy5 (and not camptothecin) was conjugated to the polymer backbone, demonstrating that the selectivity towards cancer cells is a consequence of the polymer assembly itself rather than the presence of camptothecin (Fig. S17).†

Taken together, through elegant chemistries enabled by the norbornene side chains present within the macromolecular architecture, we were able to modify our PNTC with both camptothecin and PEG to produce cytocompatible cancer-cell selective polymer–drug conjugates with controlled chemotherapeutic loading. Our results demonstrate that not only were our PNTC-*g*-CPT-*g*-PEG nanoparticles colloidally stable in physiological conditions over extended periods of time, but that their innate physicochemical properties enabled enhanced toxicity for cancerous cell lines when compared to healthy epithelial cells and fibroblasts. Through exploitation of the natural vesicular trafficking dysregulation of cancerous cells, our nanoparticles were able to accumulate substantially intracellularly, with the residence time within the cell being indicative of efficacious chemotherapeutic delivery. On the contrary, healthy cells were able to exocytose the material owing to their intact recycling pathways. These nanoparticles represent a safe an effective means to both target and deliver chemotherapeutic cargoes without need for the addition of a targeting agent.

## Conclusions

We herein report a drug–polymer conjugate that self-assembles into nanoparticles and allows for enhanced uptake and release of the anticancer drug camptothecin in cancer cells. Interestingly, nanoparticles were uptaken by both cancerous and non-cancerous cell lines, however cell viability was greatly decreased in cancer cells, while non-cancerous cells were not affected by the polymer–drug conjugate. These results were ascribed to a faster uptake of our material into cancer cells, which translates into a higher intracellular camptothecin concentration, underscoring the potential of a targeting-free approach for drug delivery to cancer. Indeed, impaired vesicular trafficking^61,62^ and higher metabolic activity^63^ are common traits of cancer cells, which lead to their higher proliferation rates compared to non-cancerous phenotypes. Both these processes contribute to a higher accumulation of nanoparticles inside cancer cells, and indeed the implication of these parameters on the efficacy and targeting of cancer therapeutics has been already exploited.^64,65^ In the case of our pH sensitive polycarbonate, the higher nanoparticle uptake in cancer cells and increased exocytosis in normal cells seem to play a key role in enhancing delivery to cancer cells. In essence, taking advantage of the kinetics of nanoparticle accumulation in cancer cells as opposed to normal cells leads to different residence times of the particles within the cells, allowing us to deliver into cancer cells more effectively. We believe that this is the first report of such kinetic control as part of a passive delivery system and as such represents a new strategy and significant advance on the state of the art, although a high selectivity for cancer cells has been previously reported when using different stimuli-responsive polymers.^66,67^ The simplicity of this approach, in combination with the biodegradable and biocompatible polymers applied opens the possibility to further explore these polymeric systems for a range of cancer therapies *in vivo*.

## Experimental section

All reagents used for the monomer synthesis, polymerisation reactions and coupling reactions were purchased from Sigma-Aldrich. All reagents were used without any further purification, except for 1,8-diazabicyclo[5.4.0]undec-7-ene (DBU) and benzyl alcohol which were dried over calcium hydride and distilled under vacuum before polymerisation. *S*-(+)-Camptothecin was purchased from Alfa Aesar. NTC,^51^ 11-azidoundecanoic acid,^68^ PEG_550_-SH,^69,70^ and CPT-azide^54,55^ were synthesized following previously published procedures. NMR spectra were recorded on a Bruker HD-300 and HD-400 spectrometer at 293 K unless stated otherwise. Chemical shifts are reported as *δ* in parts per million (ppm) and referenced to the chemical shift of the residual solvent resonances (CHCl_3_: ^1^H *δ* = 7.26 ppm, ^13^C *δ* = 77.16 ppm). Size exclusion chromatography (SEC) was used to determine the molar masses and molar mass distributions (dispersities, *Đ*_M_) of the synthesized polymers. SEC in chloroform or THF was conducted on a system comprised of a Varian 390-LCMulti detector suite fitted with differential refractive index (DRI), light scattering (LS) and ultra-violet (UV) detectors, equipped with a guard column (Varian Polymer Laboratories PLGel 5 mM, 50 × 7.5 mm) and two mixed D columns (Varian Polymer Laboratories PLGel 5 mM, 300 × 7.5 mm). The mobile phase was chloroform with 5% triethylamine eluent or THF at a flow rate of 1.0 mL min^−1^, and samples were calibrated against Varian Polymer laboratories Easi-Vials linear poly(styrene) standards (162–2.4 × 105 g mol^−1^) using Cirrus v3.3. All cell lines were purchased from ATCC.

### General procedure for the organocatalysed ROP of NTC

The ROP of NTC was carried out following a reported procedure, using 1 mol% DBU and benzyl alcohol (BnOH) in dry CDCl_3_ at ambient temperature.^51^^1^H NMR (400 MHz; CDCl_3_): *δ* 7.38 (m, 5H), 6.16 (m, 20H), 5.93 (m, 20H), 5.16 (s, 2H) 4.42 (m, 40H), 3.91–3.81 (m, 110H), 3.55 (m, 40H), 2.93 (s, 20H), 2.81 (s, 20H), 2.30 (m, 20H), 1.82 (m, 20H), 1.38–1.23 (m, 40H), 0.84–0.81 (m, 20H). *M*_n_ = 5.3 kg mol^−1^, *Đ*_M_ = 1.10 (RI detection, CHCl_3_ SEC).

### Synthesis of thiol-functionalised PEG

Synthesis of poly(ethylene glycol) with a thiol functionality was carried out as previously reported, from poly(ethylene glycol) methyl ether (molar mass *ca*. 550 g mol^−1^, 18 mmol) and 3-mercaptopropionic acid (3.82 g, 36 mmol).^51^^1^H NMR (400 MHz; CDCl_3_): *δ* 4.18 (m, 2H), 3.73–3.38 (m, 52H), 3.28 (s, 3H), 2.71–2.58 (m, 4H), 1.62 (t, 1H, ^3^*J*_H–H_ = 16). ^1^H NMR spectroscopy indicated *ca.* 98% conversion of the hydroxyl group to mercaptopropionate group. *M*_n_ = 725 g mol^−1^, *Đ*_M_ = 1.21 (RI detection, CHCl_3_ SEC).

### Synthesis of azide-functionalised camptothecin

Caution: Small organic azides are potentially explosive and must be handled with care, particularly in concentrated forms and/or in large quantities. Keep away from sources of heat, pressure, light, shocks and strong acids. *S*-(+)-Camptothecin (100 mg, 2.9 × 10^−1^ mmol), 11-azidoundecanoic acid (261 mg, 1.14 mmol), EDC·HCl (220 mg, 1.14 mmol), and DMAP (14 mg, 1.14 × 10^−1^ mmol) were dissolved in CH_2_Cl_2_ (5 mL). The mixture was stirred for 1 h at 0 °C, then cooled down to ambient temperature and stirred overnight. The residue was washed with 1 M HCl and 0.1% NaHCO_3_, then dried over MgSO_4_. The crude product was recrystallized in MeOH : CH_2_Cl_2_ (95 : 5), to yield pale yellow crystals (136 mg, 85%). ^1^H NMR (300 MHz; CDCl_3_): *δ* 8.40 (s, 1H), 8.22 (d, 1H, ^3^*J*_H–H_ = 9 Hz), 7.95 (d, 1H, ^3^*J*_H–H_ = 9 Hz), 7.84 (m, 1H), 7.67 (m, 1H), 7.22 (s, 1H), 5.68 (d, 1H, ^2^*J*_H–H_ = 18 Hz), 5.41 (d, 1H, ^2^*J*_H–H_ = 18 Hz), 3.21 (t, 2H, ^3^*J*_H–H_ = 15 Hz), 2.51–2.45 (m, 2H), 2.36–2.09 (m, 2H), 1.69–1.48 (m, 5H), 1.34–1.21 (m, 13H), 0.97 (t, 3H). ^13^C NMR (400 MHz; CDCl_3_): *δ* 172.9 (CO_lactone_), 167.7 (CO_ester_), 157.5, 152.6, 148.9, 146.3, 146.1, 131.3, 130.8, 129.8, 128.6, 128.4, 128.3, 128.1, 120.6, 96.2, 75.8, 67.2, 51.6 (CH_2_N_3 C−1_), 50.0, 33.9, 32.0, 29.5, 29.4, 29.3, 29.2, 29.1, 28.9, 26.8, 24.8, 7.7 (CH_3 C−9_). Mass spectrometry (ESI + ve); m/z = 580.25 ([M^+^ + 1] Na^+^ salt). Elemental analysis; anal. calcd for C_31_H_35_N_5_O_5_: C 66.77; H 6.33; N 12.56%. Found: C 66.21; H 6.31; N 12.35%.

### Synthesis of PNTC-*g*-CPT-*g*-PEG conjugate

In a dry vial fitted with a stirrer bar, PNTC (DP20) (50 mg, 1.09 × 10^−5^ mol) was dissolved in 500 μL of CHCl_3_. CPT-azide (21.5 mg, 3.3 × 10^−5^ mol) and/or Cy5-azide (7.8 mg, 1.3 × 10^−5^ mol) was added and the solution was stirred overnight at 90 °C. The functionalised polymer was recovered by precipitation into cold diethyl ether before being filtered and dried *in vacuo*. ^1^H NMR (500 MHz; CDCl_3_): *δ* 8.42 (s), 8.20 (d), 7.96 (d), 7.84 (t), 7.68 (t, all of them integrate for 13H), 7.21 (s, 1H), 7.36 (m, 5H), 6.15 (m), 5.93 (m, 33H with *δ* 6.15), 5.66 (d, 1H), 5.43 (d, 1H), 5.16 (m, 2H), 4.42 (m, 39H), 3.91 (m, 68H), 3.82 (m), 3.58 (m, 53H), 2.93–2.81 (m, 40H), 2.29 (m, 20H), 1.64 (m, 36H), 1.38 (m, 21H), 0.96 (t, 11H), 0.83 (m, 18H). *M*_n_ = 6.4 kg·mol^−1^, *Đ*_M_ = 1.35 (RI detection, CHCl_3_ SEC); *M*_n_ = 5.9 kg·mol^−1^, *Đ*_M_ = 1.41 (UV detection, 375 nm, CHCl_3_ SEC). PNTC-*g*-CPT (70 mg, 1.61 × 10^−5^ mol) was then re-dissolved in 500 μL of CHCl_3_. PEG_550_-SH (79.3 mg, 1.8 × 10^−5^ mol) and 2,2-dimethoxy-2-phenylacetophenone (31 mg, 1.2 × 10^−4^ mol) were added to the solution before being sealed and UV irradiated for 30 min. The functionalised polymer was recovered by precipitation into cold diethyl ether before being filtered and dried *in vacuo*. ^1^H NMR (500 MHz; CDCl_3_): *δ* 8.42, 8.21, 7.96, 7.84, 7.68 (m, CPT signals), 7.37, 5.15 (m), 4.27 (m), 3.95 (m), 3.64 (m, PEG), 3.38 (s, OC*H*_*3*_), 2.83 (m), 2.45 (m), 2.17 (m), 1.83 (m), 1.60 (m), 1.30 (m), 0.83 (m). *M*_n_ = 17.6 kg·mol^−1^, *Đ*_M_ = 1.29 (RI detection, CHCl_3_ SEC); *M*_n_ = 15.9 kg·mol^−1^, *Đ*_M_ = 1.48 (UV detection, 375 nm, CHCl_3_ SEC).

### Self-assembly and multi-angle light scattering analysis

PNTC-*g*-PEG polymers were self-assembled by a solvent switch method. A solution of polymer was dissolved in THF at a concentration of 5 mg mL^−1^ and stirred overnight. 9 mL of DI H_2_O was then added slowly (0.6 mL h^−1^). Polymer solutions were stirred at room temperature overnight before being filtered (0.45 μm filters) and analysed *via* SLS, DLS, TEM, and cryo-EM. PNTC-*g*-CPT-*g*-PEG particles prepared with this method were then treated with acidic conditions (0.01M HCl) and analysed *via* SLS.

### Cryo-EM imaging

Cryo transmission electron microscopy (Cryo-EM) analysis was performed on a JEOL 2200 FX microscope. Samples were deposited onto plasma cleaned lacey carbon grids at a concentration of 30 mg mL^−1^. Grid were then blotted for 3s and immersed in liquid ethane prior to be inserted in the cryo holder for microscopic analysis.

### Viability studies

A549, PC3, and CHO-K1 cell lines were cultured in F12K media with the addition of 10% FBS and 100U mL^−1^ pen/strep. MCF-7, IMR-90, 3T3, and Hs 792 were cultured in DMEM with the addition of 10% FBS and 100U mL^−1^ pen/strep. Media for MCF-7 was enriched with 0.01 mg mL^−1^ human recombinant insulin. MDA-MB-468 were cultured in Leibovitz's L-15 medium with the addition of 10% FBS and 100U mL^−1^ pen/strep. Cells were seeded on 12 well plates at 2000 cells cm^−2^ and left adhere and proliferate for 72 h. The medium was then replaced with PNTC-*g*-PEG, PNTC-*g*-CPT-*g*-PEG, or CPT-azide in a concentration range from 1 to 10 mg mL^−1^ for PNTC-*g*-PEG and from 0 to 100 μM for the free drug and polymer–drug conjugate (relative to concentration of camptothecin). After 72 h the solution was removed, cells were washed with PBS (1 mL × 3) and incubated with MTS cell viability assay for 2–4 hr, following manufacturer's instructions. 100 μL of solution was taken from each well and placed in triplicate in a 96-well plate. The fluorescence intensity (FI) was detected in Plate Reader by excitement at 530 nm and emission at 590 nm. Cell data were reported as viability % in comparison with control sample. Significance was set at a *p*-value ≤ 0.05. Experiments were performed in triplicate (3 repeats per experiment and 3 independent experiments per cell line). IC50 and 95% confidence interval for all data was calculated using Graphpad Prism 8 software.

### Live cell imaging

Cells were imaged using a Zeiss 710 laser scanning confocal microscope (Carl Zeiss Microscopy, Oberkochen, Germany) housed within the Australian National Fabrication Facility (ANFF) Queensland Node. This machine is equipped with a heated stage, 20× and 40× water objectives, a 405-nm diode, and helium–neon and argon lasers. Prior to imaging, cells in 1.5 mL of fresh medium were plated into slide-bottomed culture dishes (Mattek), returned to the incubator and left to grow overnight. Cells were then incubated with the polymer-CPT conjugate labelled with Cy5 and the fluorescence was monitored over time (633 nm laser). Fluorescence intensities at each time point were obtained by processing each image (at least 10 images per time point) using the region of interest tool within the ZEN Zeiss lite software (Carl Zeiss Microscopy, Oberkochen, Germany). Retrieved values were transferred to Microsoft Excel for further analysis.

## Author contributions

The manuscript was written through contributions of all authors. All authors have given approval to the final version of the manuscript.

## Conflicts of interest

There are no conflicts of interest to declare.

## Supplementary Material

BM-011-D2BM01626E-s001
