# Supplementary material for: Enhanced drug delivery to cancer cells through a pH-sensitive polycarbonate platform
Source: Biomater Sci. 2022 Dec 15;11(3):908–15. doi: 10.1039/d2bm01626e (PMC9890502; doi:10.1039/d2bm01626e)
Supplement: BM-011-D2BM01626E-s001 [file BM-011-D2BM01626E-s001.pdf]

Electronic Supplementary Material (ESI) for Biomaterials Science.
This journal is © The Royal Society of Chemistry 2022

Supplementary information

Enhanced drug delivery to cancer cells through a pH-sensitive polycarbonate platform

Maria C. Arno[a,b], Joshua D. Simpson[c,d,e], Lewis D. Blackman[f], Ruairí P. Brannigan[f], Kristofer J. Thurecht[c,d,e], Andrew P. Dove[a]**

a. School of Chemistry, University of Birmingham, Edgbaston, Birmingham, B15 2TT, United Kingdom
b. Institute of Cancer and Genomic Sciences, University of Birmingham, Edgbaston, Birmingham, B15 2TT, United Kingdom
c. Australian Institute for Bioengineering and Nanotechnology, The University of Queensland, St. Lucia, Queensland 4072, Australia
d. Centre for Advanced Imaging, The University of Queensland, St. Lucia, Queensland 4072, Australia
e. ARC Centre of Excellence in Convergent Bio-Nano Science and Technology, The University of Queensland, St. Lucia, Queensland 4072, Australia
f. Department of Chemistry, The University of Warwick, Coventry CV4 7AL, United Kingdom

Supplementary data

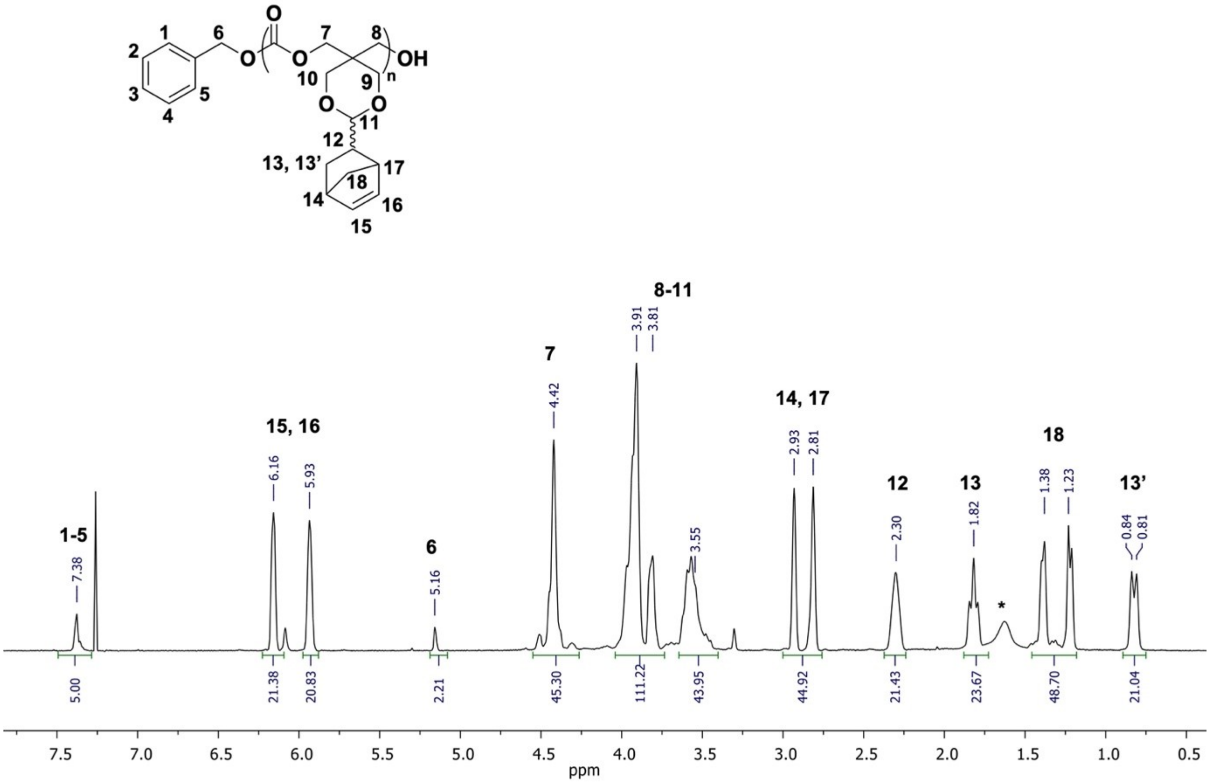

Figure S1. ^{1}H NMR spectrum of poly(NTC) (PNTC) (DP20) initiated from benzyl alcohol (400 MHz, $CDCl_3$). * H_2O.

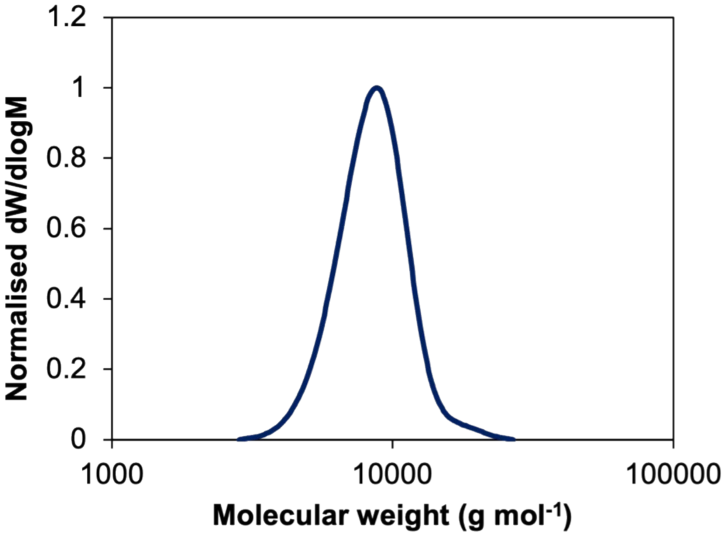

Figure S2. Size exclusion chromatograms of PNTC (DP 20), RI detection. Eluent: $CHCl_3$, standard: PS.

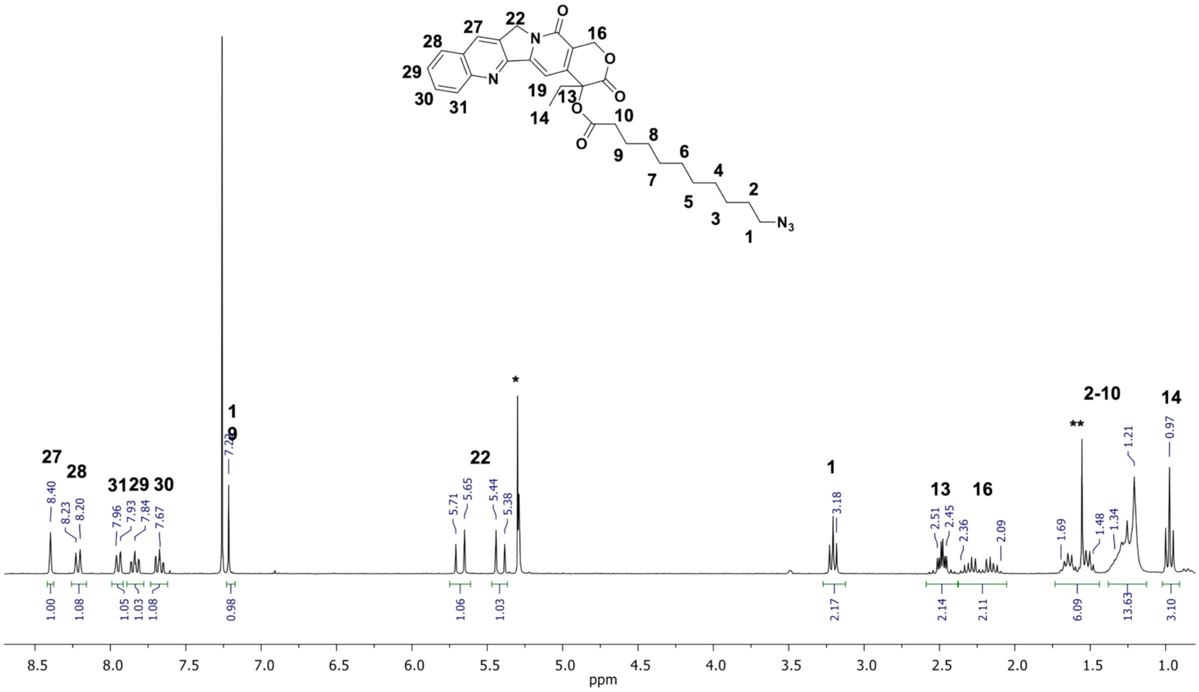

Figure S3. ^{1}H NMR spectrum of CPT-azide (400 MHz, $CDCl_3$). * CH_2Cl, ** H_2O.

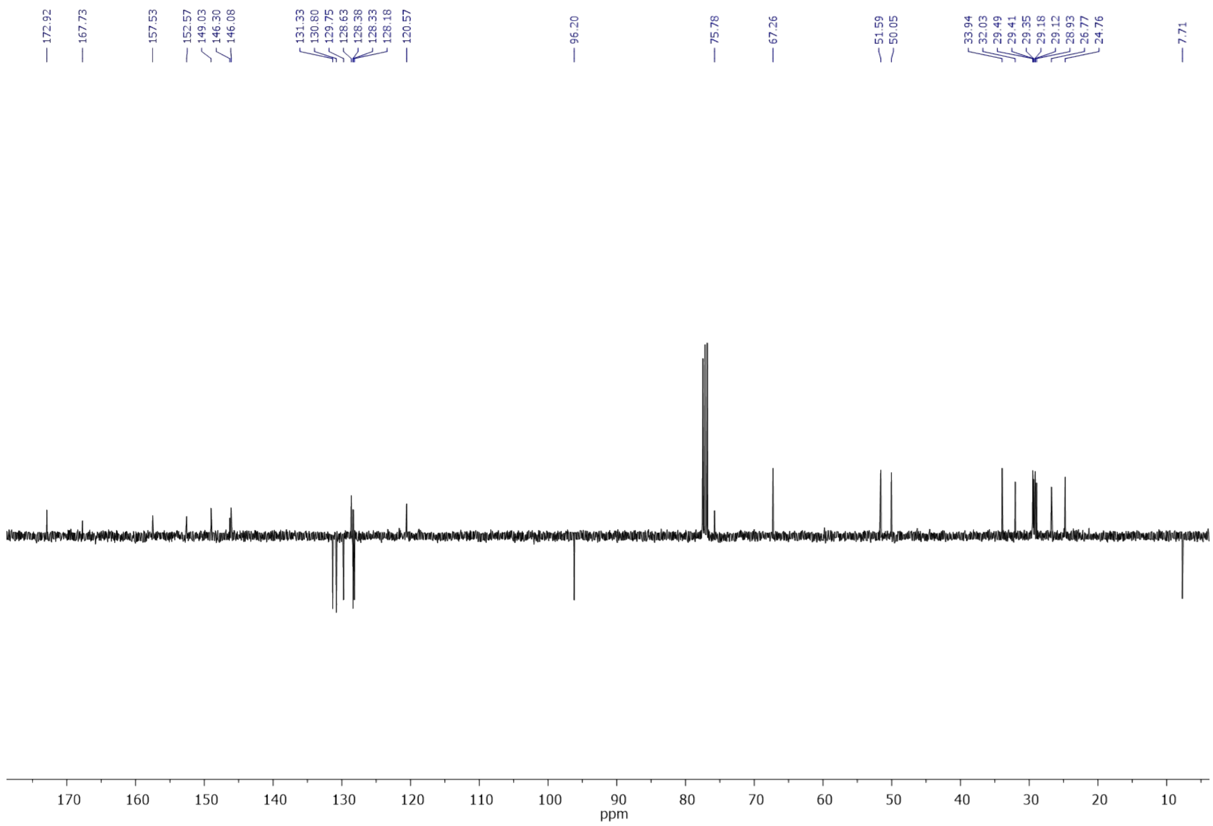

Figure S4. ^{13}C NMR spectrum of CPT-azide (400 MHz, $CDCl_3$).

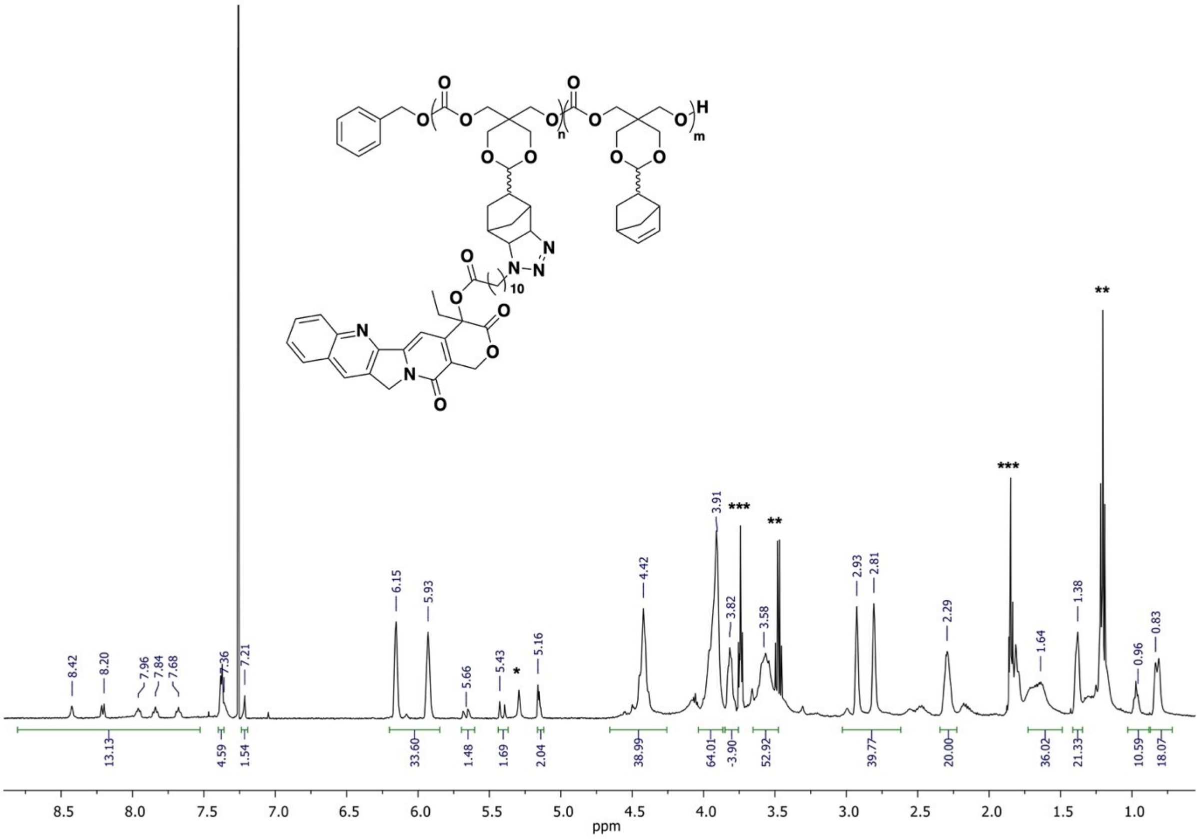

Figure S5. ^{1}H NMR spectrum of PNTC$_{20}$-*g*-CPT (500 MHz, $CDCl_3$). Integration of the aromatic region for camptothecin protons indicates a 15% functionalization (13 proton/5 = 2.6 units of CPT per polymer chain). * CH_2Cl, ** Et_2O, *** THF.

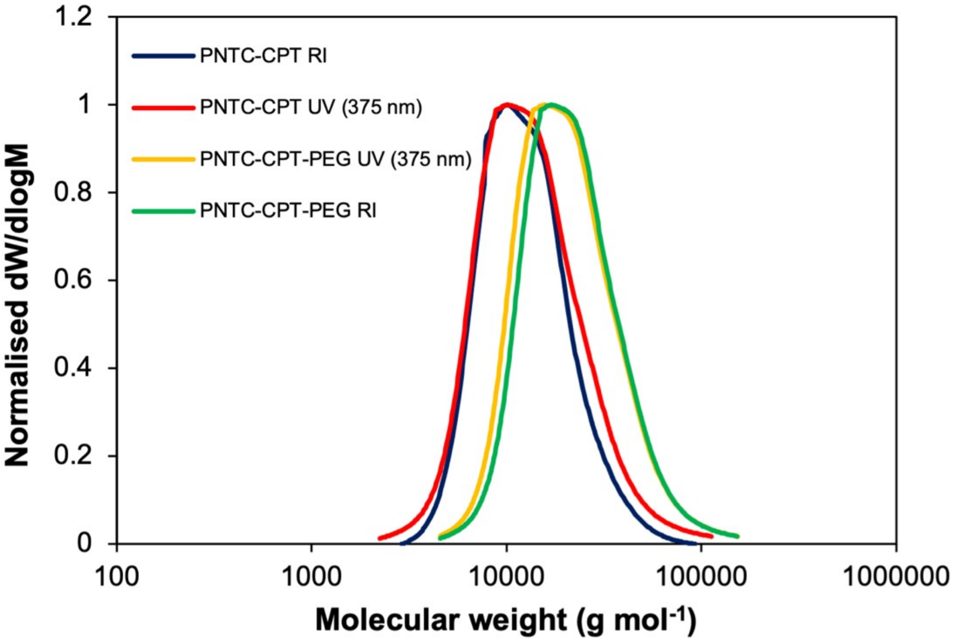

Figure S6. Size exclusion chromatograms of PNTC-*g*-CPT conjugate (RI and UV detection) and PNTC-*g*-CPT-*g*-PEG (RI and UV detection). Eluent: THF, standard: PS.

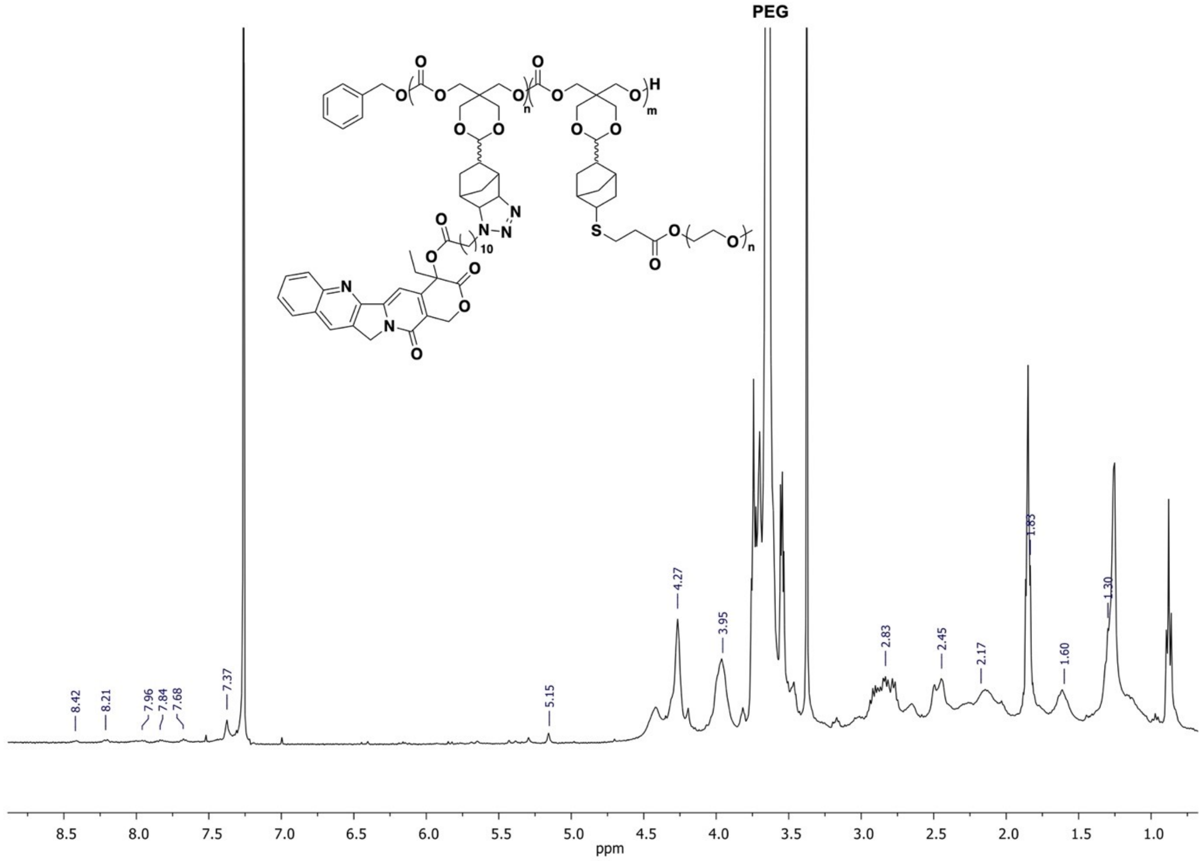

Figure S7. ^{1}H NMR spectrum of PNTC-*g*-CPT-*g*-PEG (500 MHz, $CDCl_3$).

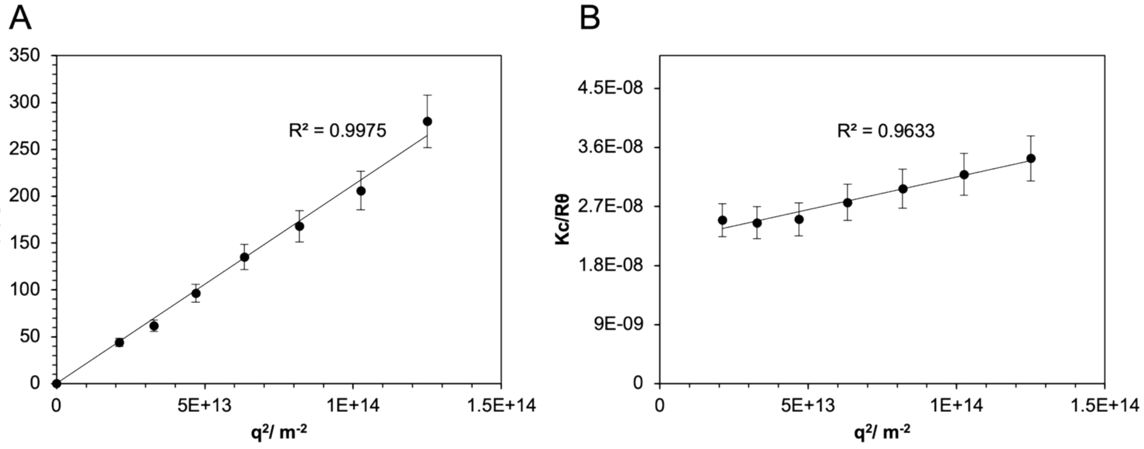

Figure S8. Multi-angle light scattering analysis of PNTC-*g*-CPT-*g*-PEG_{550} assemblies in water at pH 7 (1 mg/mL). A) shows multiple angle DLS data and B) shows the partial Zimm plot from SLS analysis (R_g/R_h = 0.992).

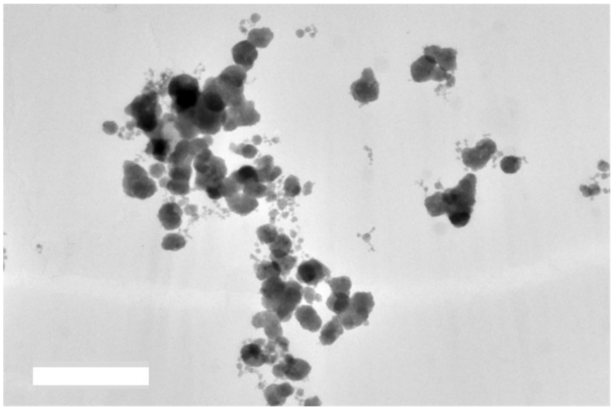

Figure S9. TEM micrograph of PNTC-g-CTP-g-PEG assemblies in water. 1% uranyl acetate was used as negative stain. Scale bar = 500 nm.

Table S1. Structural parameters of PNTC-*g*-CPT-*g*-PEG assemblies in water at different pH (1 mg mL^{-1}) analysed by Multi Angle DLS and SLS.

pH	N_{agg}	R_g/R_h	R_h (nm)
**7.4**	1550	0.99	104
**6.0**	1190	1.01	161
**5.0**	4260	2.52	112
**4.0**	4230	2.12	131

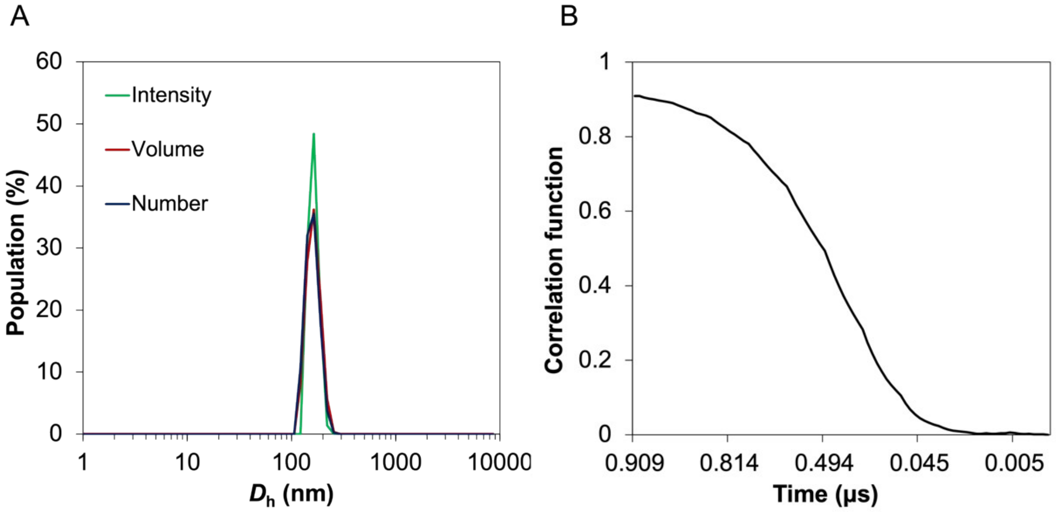

Figure S10. Size distribution (A) and correlation function (B) from DLS analysis show only one particle distribution for PNTC-*g*-CPT-*g*-PEG assemblies after being suspended in DMEM with 10% FBS for 72 h.

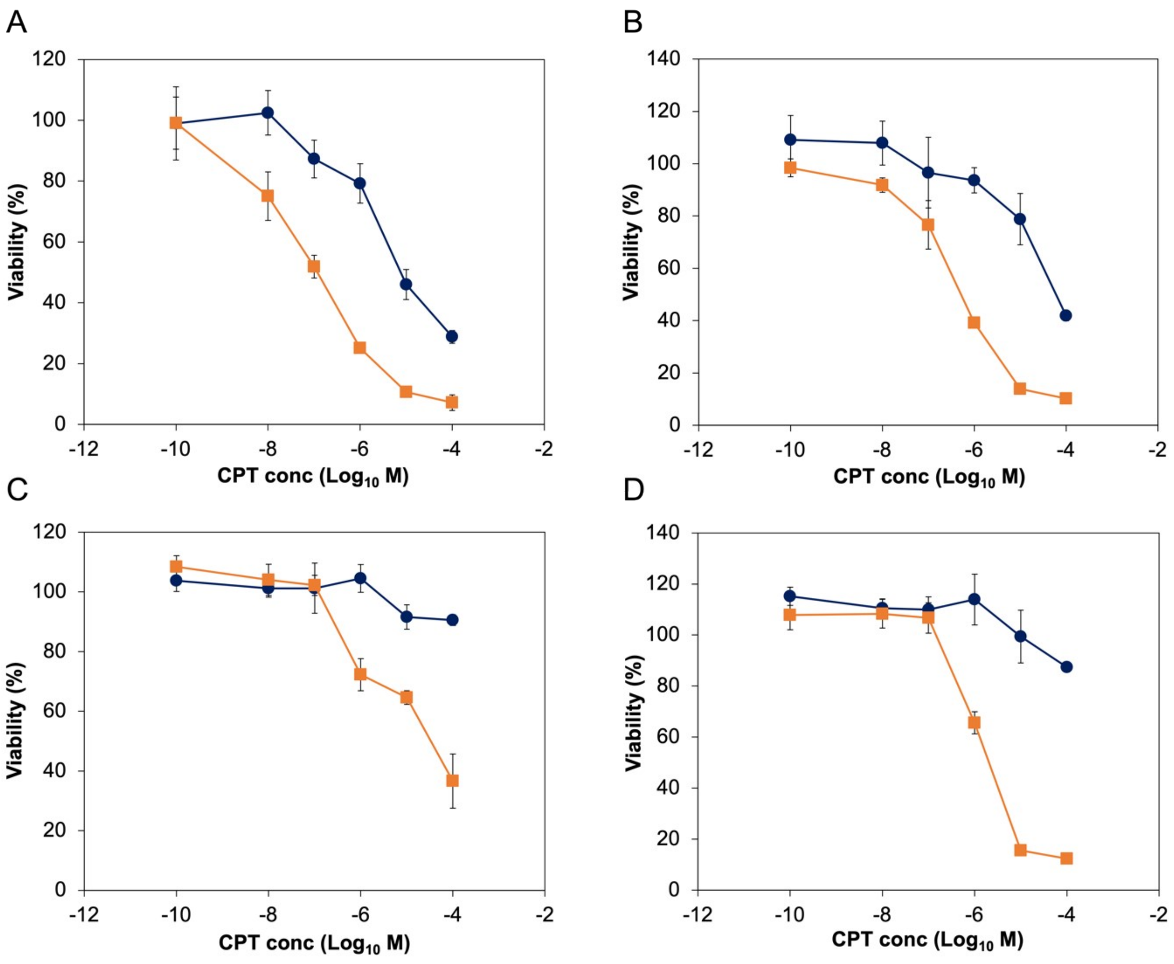

Figure S11. Viability of MDA-MB-468 (A) and MCF-7 (B) cancer cell lines, and 3T3 (C), and CHO-K1 (D) non-cancerous cell lines when incubated for 72 h with camptothecin azide (orange line, squared marker), PNTC-g-CPT-g-PEG (dark blue line, round marker).

Table S2. IC50 and 95% confidence intervals for cancerous cell lines incubated with camptothecin (CPT) azide and the drug-polymer conjugate.

Cell type	IC50 CPT azide alone (µM)	95% confidence interval (µM)	IC50 drug-polymer conjugate (µM)	95% confidence interval (µM)
A549	0.184	1.4×10^{-2} and 2.4	3.06	6.2×10^{-1} and 15.1
PC3	0.068	8.2×10^{-3} and 5.6×10^{-1}	4.48	6.4×10^{-1} and 31.3
MCF-7	0.44	7.9×10^{-2} and 2.5	6.29	1.1 and 36
MDA-MB-468	0.082	2.3×10^{-2} and 2.9×10^{-1}	3.82	5.1×10^{-1} and 28.7

Table S3. IC50 and 95% confidence intervals for non-cancerous cell lines incubated with camptothecin (CPT) azide.

**Cell type**	**IC50 CPT azide alone (μM)**	**95% confidence interval (μM)**
IMR-90	0.096	4.1×10^{-3} and 2.3
HS792	0.487	2×10^{-2} and 11.6
CHOK-1	0.28	7.1×10^{-2} and 1.1
NIH-3T3	9.8	1.2×10^{-1} and 80.3

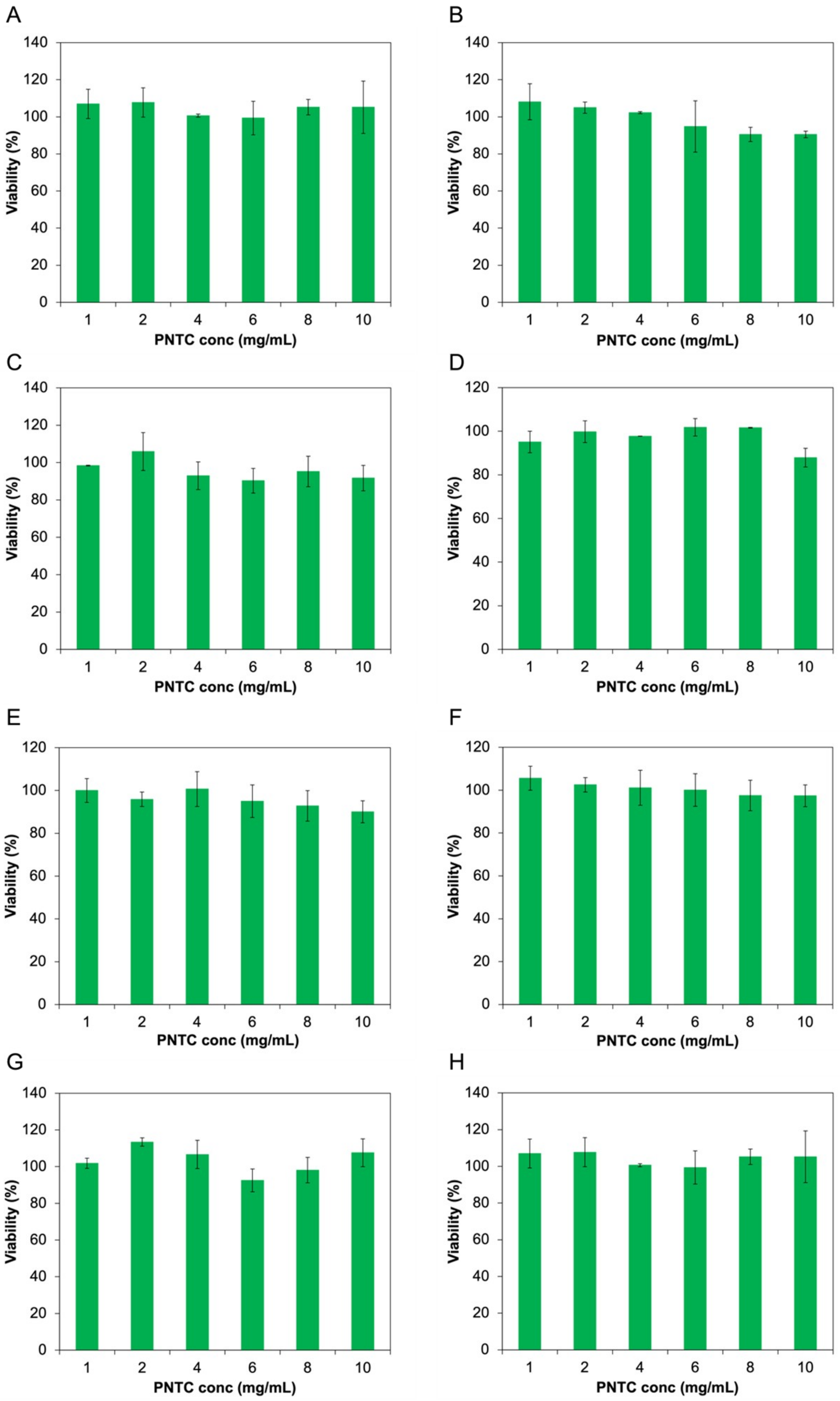

Figure S12. Viability of A549 (A), PC3 (B), MDA-MB-468 (C), MCF-7 (D) cancer cell lines, and IMR-90 (E), CHO-K1 (F), 3T3 (G), and HS792 (H) non-cancerous cell lines when incubated for 72 h with PNTC-*g*-PEG.

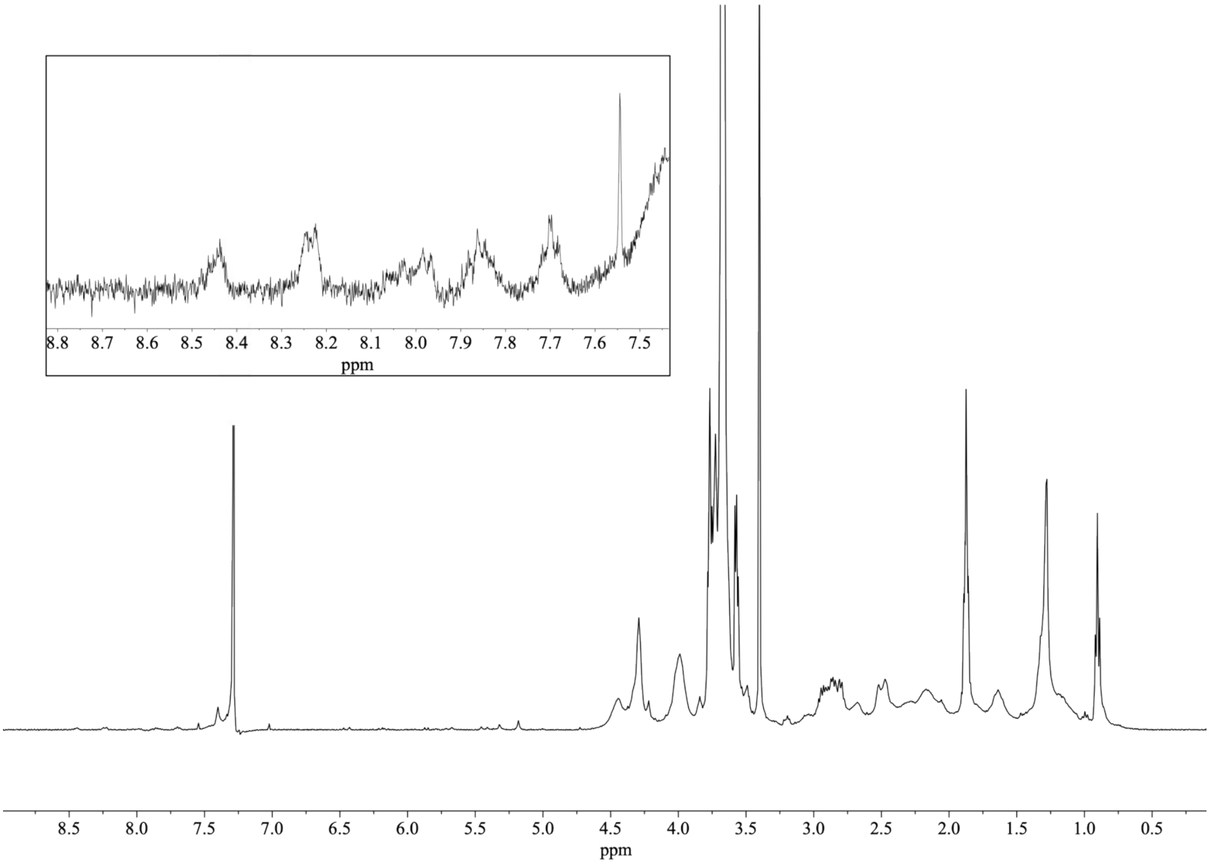

Figure S13. ^{1}H NMR spectrum of PNTC-*g*-Cy5-*g*-CPT-*g*-PEG (500 MHz, $CDCl_3$).

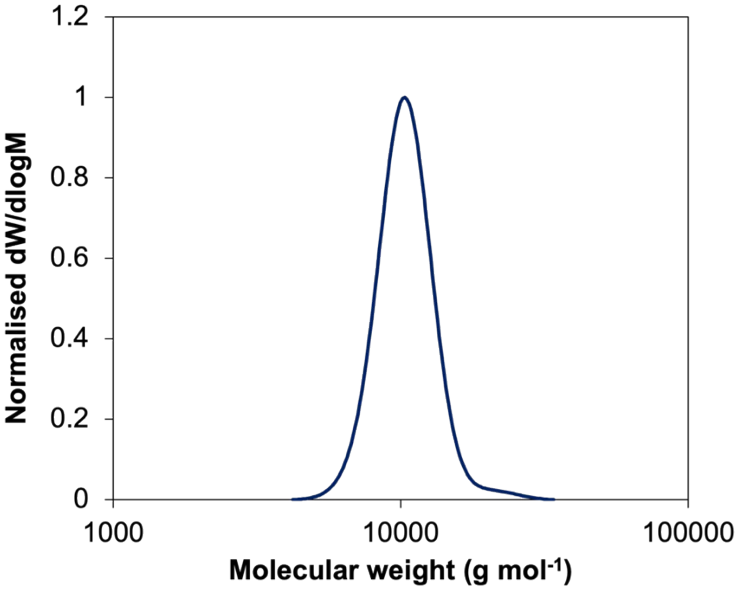

Figure S14. Size exclusion chromatogram of PNTC-*g*-Cy5-*g*-CPT-*g*-PEG conjugate (RI detection). Eluent: $CHCl_3$, standard: PS.

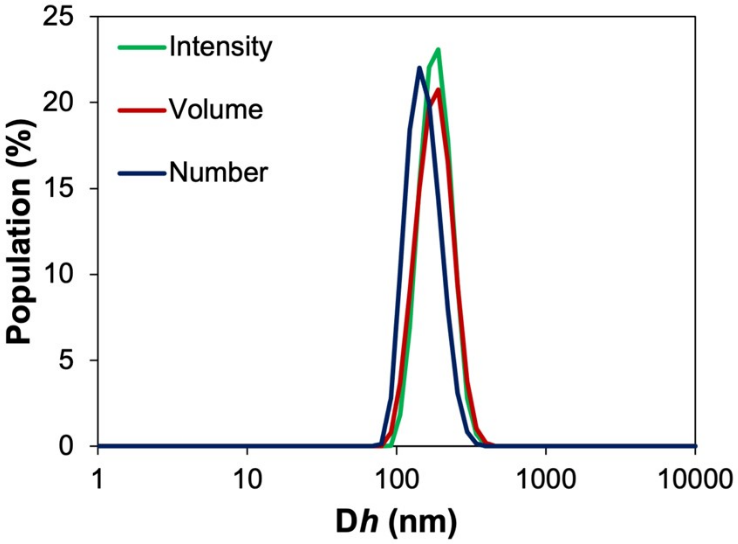

Figure S15. Size distribution from DLS analysis of PNTC-*g*-Cy5-*g*-CPT-g-PEG nanoparticles.

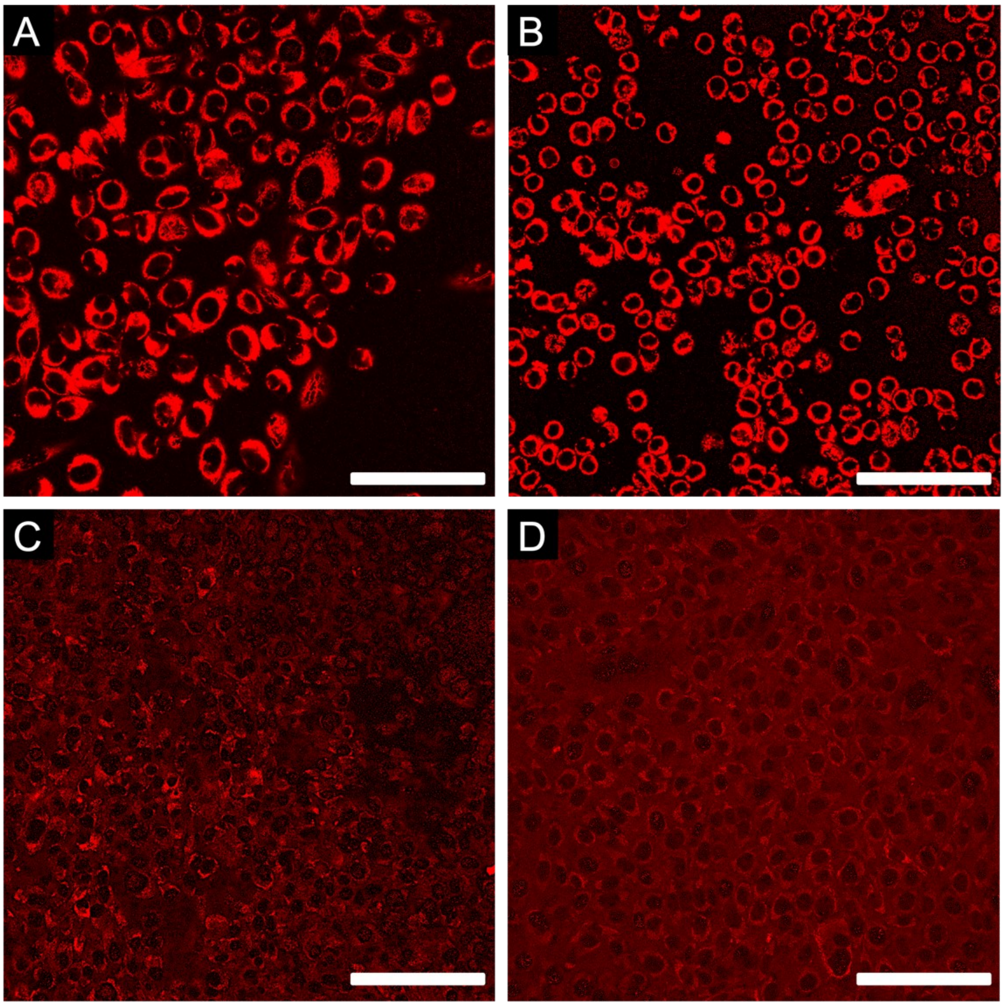

Figure S16. Confocal fluorescent images of PC3 (A), MDA-MB-468 (B), 3T3 (C), and CHO-K1 (D) cells incubated with PNTC-*g*-CPT-*g*-Cy5-*g*-PEG for 24 h. Scale bar = 10 µm.

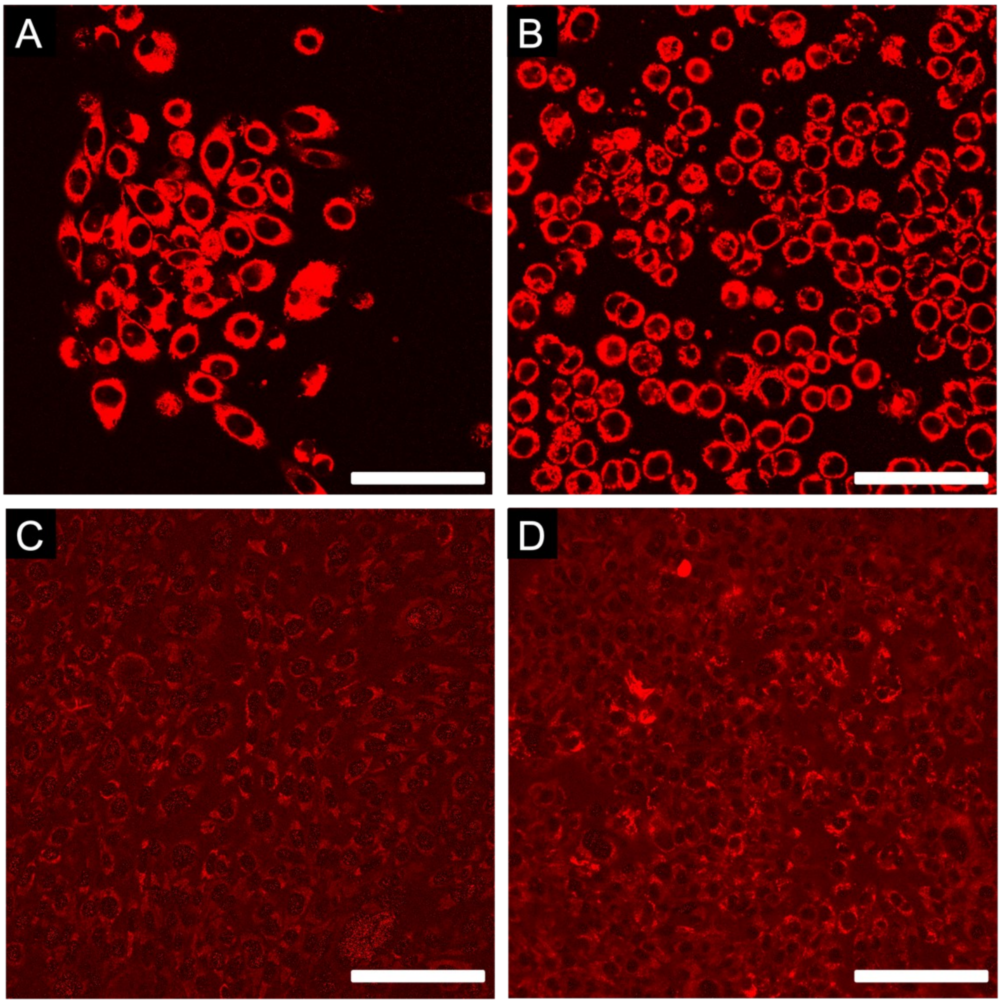

Figure S17. Confocal fluorescent images of PC3 (A), MDA-MB-468 (B), 3T3 (C), and CHO-K1 (D) cells incubated with PNTC-*g*-Cy5-*g*-PEG for 24 h. Scale bar = 10 μm.
